# Left superior segmentectomy for a patient with displaced anomalous apicoposterior branches of the pulmonary vein and bronchus: a case report

**DOI:** 10.1186/s40792-020-01097-0

**Published:** 2021-01-06

**Authors:** Hiroki Matsumoto, Hidemi Suzuki, Takahide Toyoda, Terunaga Inage, Kazuhisa Tanaka, Yuichi Sakairi, Fumihiro Ishibashi, Takahiro Nakajima, Ichiro Yoshino

**Affiliations:** Department of General Thoracic Surgery, Chiba University Graduate School of Medicine, 1-8-1, Inohana, Chuo-ku, Chiba, 260-8670 Japan

**Keywords:** Lung cancer, Displaced bronchus, Segmentectomy, Anomalous pulmonary vein, Three-dimensional computed tomography

## Abstract

**Background:**

Knowledge of anatomical abnormalities and variations in pulmonary vessels and bronchi is critical for patients requiring a lung segmentectomy. To the best of our knowledge, this is the first case of a tumor existing in the lower lobe in conjunction with a displaced B^1+2^ in which the B^1+2^ was not accidentally cut during surgery.

**Case presentation:**

A 71-year-old woman was referred to our hospital after a part-solid lung cancer was found in the superior segment of her left lung on chest computed tomography. Preoperative three-dimensional computed tomography revealed a displaced anomalous left B^1+2^ arising from the left main bronchus and anomalous V^1+2^ returning to the inferior pulmonary vein. We identified these anomalies during surgery and performed a left superior segmentectomy. After an unremarkable recovery, the patient was discharged from the hospital on the eighth day postoperative.

**Conclusions:**

We used a three-dimensional construction system during the preoperative planning of the pulmonary segmentectomy to better understand the bronchovascular structures. When performing surgery where anatomical abnormalities are present, there is the possibility of misidentification. Using the three-dimensional construction system, it was possible to perform safer surgery, as the surgeons were able to preoperatively prepare for any abnormalities.

## Background

Most bronchial abnormalities are found in the right upper lobe of the lung; however, abnormalities have also been reported in the left upper lobe [1, 2]. Thin-sliced computed tomography (CT) provides detailed images of the segmental bronchovascular structures of the lung, and three-dimensional reconstruction of CT imaging data allows for a better understanding of the spatial relationships of the segmental branches. We report the case of a patient diagnosed with a part-solid lung cancer in her lower left lobe and with a displaced apicoposterior branch of the bronchus (B^1+2^) and vein (V^1+2^). The patient underwent a left superior segmentectomy (S^6^). The patient’s anatomy was well understood preoperatively due to the use of three-dimensional CT images. Few reports exist of patients with lung cancer with a displaced B^1+2^. In some of these reports, the displaced B^1+2^ was accidentally cut by a stapler during separation of the interlobar fissure; however, the bronchus did not need to be reconstructed. To the best of our knowledge, this is the first case of a tumor existing in the lower lobe in conjunction with a displaced B^1+2^. If anatomical abnormalities are not known preoperatively, they may be mistakenly cut and the wrong lung segmentectomy may be performed.

## Case presentation

**Fig. 1:**
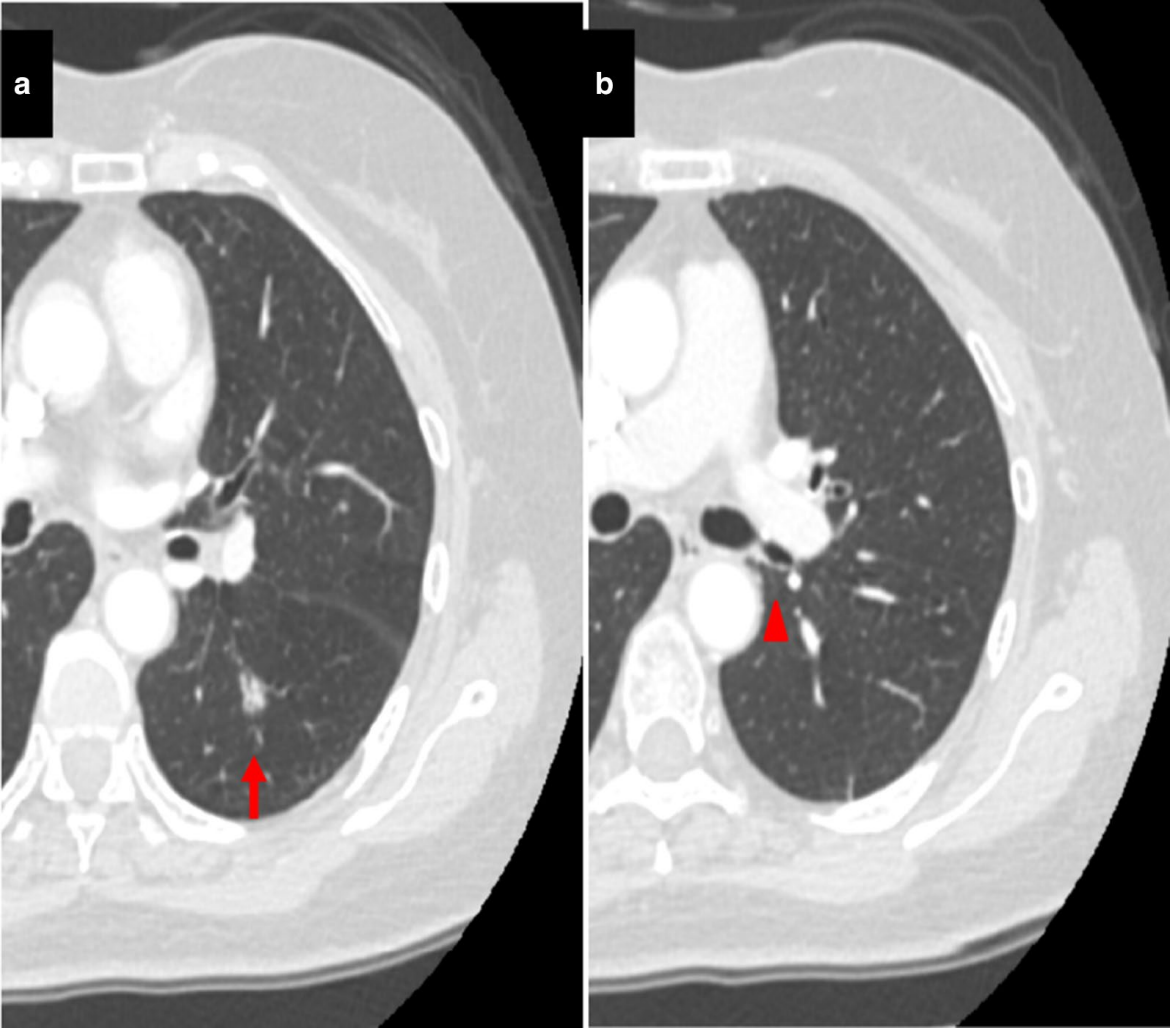
Chest computed tomography images. Contrast-enhanced CT imaging shows the lung tumor located at the left S6 segment (**a**, arrow) and a displaced B^1+2^ (**b**, arrowhead)

A 71-year-old female who was recently diagnosed with a lung nodule presented to our department. The nodule was found on a chest CT initially performed to screen for recurrence of previously treated breast cancer. The nodule was located in the left superior lung segment (S^6^) and was characterized as a part-solid tumor measuring 1.2 cm. Preoperative contrast-enhanced CT imaging showed the apicoposterior bronchus (B^1+2^) arising from the left main bronchus behind the left main pulmonary artery, and the apicoposterior vein (V^1+2^) draining into the left inferior pulmonary vein (Fig. 1).

**Fig. 2:**
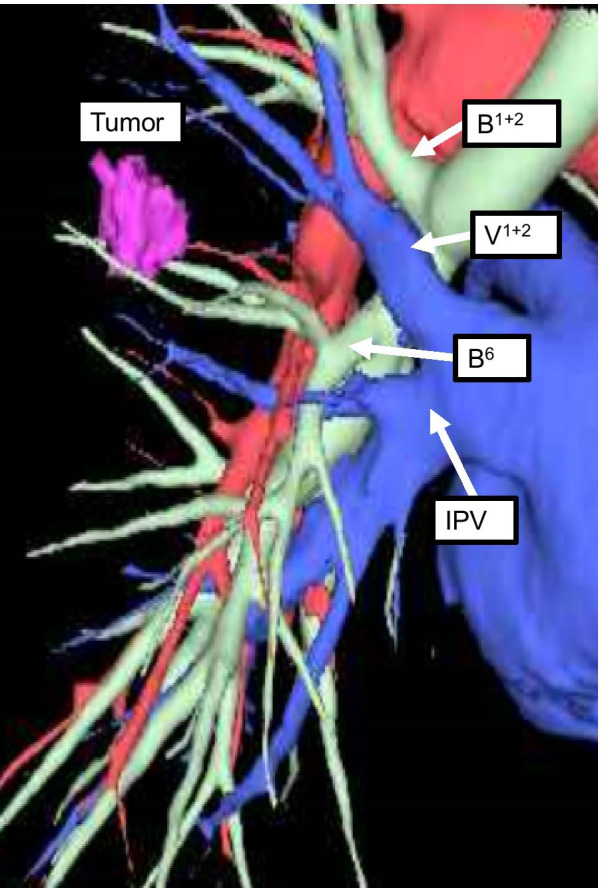
A three-dimensional reconstructed image. SYNAPSE VINCENT was used to preoperatively construct a three-dimensional image of the patient’s anatomy. *IPV* inferior pulmonary vein

A three-dimensional construction system (SYNAPSE VINCENT, Fujifilm Medical, Tokyo, Japan) was used to reconstruct the CT images to better understand the spatial relationship of the bronchovascular structures preoperatively. The B^1+2^ and V^1+2^ were clearly recognized at the interlobar fissure and located near the segmental bronchovascular structures that were to be resected (Fig. 2).

**Fig. 3:**
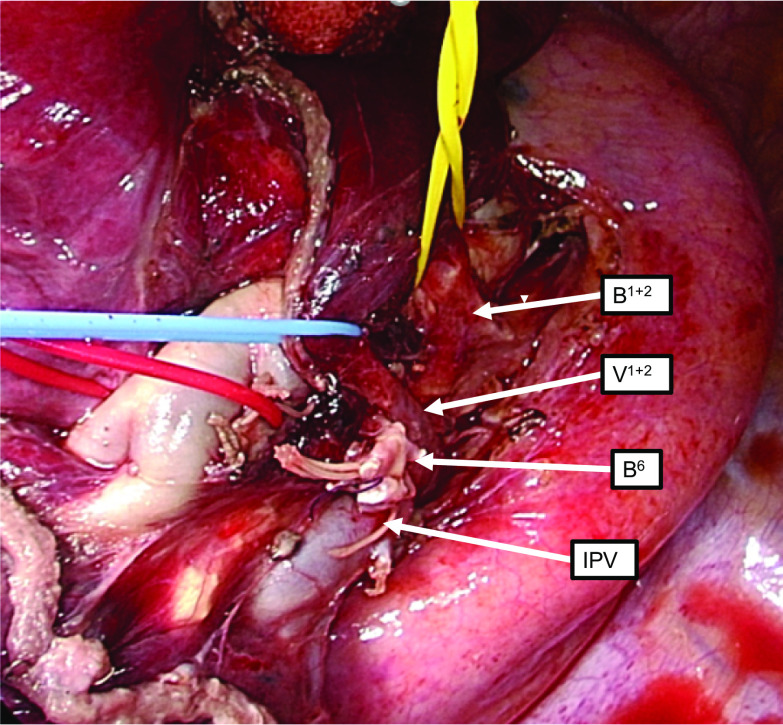
Thoracoscopic view. Intraoperative findings after segmentectomy. *IPV* inferior pulmonary vein

The left superior segmentectomy was performed through a 10-cm axial incision. Lung parenchymal fusion was observed between S^1+2^ and S^6^. The displaced V^1+2^ and B^1+2^ were easily identified posterior to the hilum and were separately taped posterior to the main pulmonary artery. Next, A^6^ was identified at the fissure between S^1+2^ and S^6^, and the fissure and artery were divided. Then, B^6^ was exposed and divided. The remaining lung tissue between S^6^ and S^8−10^ was divided using an automated stapler, and the S^6^ segmentectomy was successfully completed (Fig. 3).

The total operation time was 235 min, and the estimated blood loss was 70 mL. The pathological diagnosis was an invasive mucinous adenocarcinoma with a 15-mm nodule. The tumor's surgical margins were negative. The patient was discharged from the hospital on postoperative day 8 after an unremarkable recovery. The patient provided informed consent for publication of this case report.

## Discussion

Tracheobronchial anomalies are classified as either supernumerary bronchi or displaced bronchi [3]. The incidence of tracheobronchial anomalies has been reported as 0.64–0.76%, and 75–89% of these anomalies are located in the right upper lobe [1, 2]. This case is similar to “Left B^1+2^ Type” described by Yaginuma et al. There were incomplete lobulations between the S^1+2^ and S^6^, the main pulmonary artery passed in front of the B^1+2^, and V^1+2^ joined inferior pulmonary vein[2]. Shiina et al. revealed that variant-type pulmonary vein anomalies are more common in the right lung (32.8% of all pulmonary vein anomalies) than in the left lung (2.6%) [4].

A displaced bronchus or displaced V^1+2^ in the left upper lung lobe is rare. Preoperative, three-dimensional, multi-dissector CT angiography allows visualization of pulmonary vasculature and bronchi anatomy. Akiba et al. recommended the use of this technology for surgical planning in patients undergoing an anatomical resection due to lung cancer [6]. Ohtaka et al. described that VATS segmentectomy was performed for a lung abscess patient with a displaced subsegmental bronchus and recommended a preoperative 3D CT may be helpful for identifying anatomical anomalies [7].

To the best of our knowledge, only seven reports exist of patients with lung cancer with a displaced B^1+2^. In each of these patients, a tumor was found in the upper lobe: five underwent a lobectomy or pneumonectomy, and two underwent an S^1+2^ segmentectomy. In this study, our patient underwent an S^6^ segmentectomy. For these procedures, the anomalous branches of the pulmonary structures must be identified and preserved (Table 1) [7–14]. The recognition of such anomalies is critical in patients undergoing not only a left upper lobectomy, superior segmentectomy, S^1+2^ segmentectomy but also a left lower lobectomy or superior segmentectomy; this is especially important for the separation of the interlobar fissure between S^1+2^ and S^6^. In two of the previously reported cases, the displaced B^1+2^ was accidentally cut by a stapler during separation of the interlobar fissure [9, 10]. However, the bronchial structure did not require repair in those cases because a left upper lobectomy was performed.

**Table 1:** Reports of lung resection for lung cancer in patients with displaced B^1+2^

First author	Year	Age/sex	Procedure	Anomalous PV
Motohashi	1995	52/F	LP	None
Shimamoto	2008	81/F	S^1+2^ Seg	Unknown
Tsukioka	2011	62/F	LUL	Unknown
Ikuta	2013	83/M	LUL	V^1+2^
Asakura	2013	52/M	LUL	Unknown
Onuki	2016	83/F	LUL	None
Yanagiya	2020	70/M	S^1+2^ Seg	Unknown
Present case	2020	71/F	S^6^ Seg	V^1+2^

*PV* pulmonary vein, *M* male, *F* female, *LP* left pneumonectomy, *LUL* left upper lobectomy, *Seg* segmentectomy

In our patient, the displaced B^1+2^ and V^1+2^ were easily preserved, and an S^6^ segmentectomy was safely achieved. If we had not used preoperative three-dimensional reconstruction, B^1+2^ and V^1+2^ may have been misidentified as B^6^ and V^6^. Misidentification may have led to them being mistakenly cut, which may have gone unnoticed during the operation.

## Conclusions

We successfully performed a left S^6^ segmentectomy for lung cancer by preserving the displaced B^1+2^ and V^1+2^. This was possible due to the use of three-dimensional CT during the preoperative planning process.

## Data Availability

All data supporting the conclusions of this article are included within the published article.

## Abbreviations

CT: Computed tomography
CT: Computed tomography

## References

[1] Ohta S, Saito Y, Usuda K, Kanma K, Sagawa M, Sato M (1986). Tracheobronchial anomalies: report of 71 cases. J Jpn Soc Resp Endosc.

[2] Yaginuma H (2020). Investigation of displaced bronchi using multidetector computed tomography: associated abnormalities of lung lobulations, pulmonary arteries and veins. Gen Thorac Cardiovasc Surg.

[3] Foster-Carter AF (1946). Broncho-pulmonary abnormalities. Br J Tuberc Dis Chest.

[4] Shiina N, Kaga K, Hida Y, Sasaki T, Hirano S, Matsui Y (2018). Variations of pulmonary vein drainage critical for lung resection assessed by three-dimensional computed tomography angiography. Thorac Cancer.

[5] Akiba T, Marushima H, Harada J, Kobayashi S, Morikawa T (2008). Anomalous pulmonary vein detected using three-dimensional computed tomography in a patient with lung cancer undergoing thoracoscopic lobectomy. Gen Thorac Cardiovasc Surg..

[6] Ohtaka K, Iwashiro N, Watanabe K, Mizota T, Takahashi R, Suzuoki M (2019). A left lung abscess with a displaced subsegmental bronchus and anomalous pulmonary artery and vein: a case report. Surg Case Rep..

[7] Motohashi S, Yamaguchi Y, Takeda T, Aoyagi H, Ohtsuka T, Yokosuka T (1995). A case of lung squamous cell carcinoma in anomalously situated subsegmental bronchus of left upper lobe; a case report. Jpn J Chest Surg.

[8] Shimamoto A, Takao M, Kodama H, Murashima S, Shomura S, Tarukawa T (2008). A case of left apicoposterio segmentectomy for lung cancer occurring in a displaced anomalous bronchus. J Jpn Soc Resp Endosc.

[9] Tsukioka T, Yamamoto R, Takahama M, Nakajima R, Tada H (2011). A case of lung cancer arising from abnormal bronchi. J Jpn Assoc Chest Surg.

[10] Ikuta Y, Tamura K, Sakamoto A, Hidaka K (2013). Lung cancer in the left upper lobe with a displaced anomalous left B^1+2^ bronchus accompanied by an anomalous V^1+2^ pulmonary vein: a surgical case. Jpn J Chest Surg.

[11] Asakura K, Imanishi N, Matsuoka T, Nagai S, Matsuoka K, Ueda M (2014). Video-assisted thoracic surgery lobectomy for lung cancer with displaced b(1+2). Ann Thorac Cardiovasc Surg..

[12] Onuki T, Ueda S, Yamaoka M, Inagaki M (2016). Displaced B1+2 found at video assisted thoracic surgery for lung cancer of left upper lobe. Kyobu Geka.

[13] Hayashi K, Motoishi M, Horimoto K, Sawai S, Hanaoka J (2018). Left upper division segmentectomy with a simultaneous displaced bronchus and pulmonary arteriovenous anomalies: a case report. J Cardiothorac Surg.

[14] Yanagiya M, Yamaguchi H, Hiyama N, Matsumoto J (2020). Left apicoposterior segmentectomy for lung cancer with displaced segmental bronchus: a case report. J Cardiothorac Surg.

